# Physical barriers for suppression of movement of adult stink bugs into cotton

**DOI:** 10.1007/s10340-014-0564-8

**Published:** 2014-02-08

**Authors:** P. Glynn Tillman

**Affiliations:** Crop Protection and Management Research Laboratory, USDA, ARS, PO Box 748, Tifton, GA 31793 USA

**Keywords:** Barrier wall, Sorghum sudangrass, Buckwheat, Nectar provision

## Abstract

*Nezara viridula* (L.), *Euschistus servus* (Say), and *Chinavia hilaris* (Say) (Heteroptera: Pentatomidae) are economic pests of cotton in the southeastern USA. Because adult stink bugs exhibit edge-mediated dispersal at crop-to-crop interfaces as they colonize cotton, strategic placement of physical barriers at these interfaces could manage these pests. The objective of this study was to determine the effectiveness of a physical barrier, either synthetic or plant-based, at the peanut-to-cotton interface for suppressing stink bugs that would move to cotton. In 2012 and 2013, sorghum sudangrass (2.4 and 2.1 m high, respectively) was significantly taller than cotton (1.4 and 1.3 m high, respectively) which was taller than peanut (0.4 and 0.5 m high, respectively). Buckwheat (0.6 m high), planted only in 2012, was significantly taller than peanut, but shorter than cotton. For both years of the study, sorghum sudangrass and a 1.8-m-high polypropylene barrier wall effectively deterred dispersal of stink bugs into cotton. Because each of these barriers was taller than cotton, their success in protecting cotton likely was due to disruption of the flight of stink bugs from low-growing peanut into cotton. The shortest barrier wall (0.6-m-high) did not suppress stink bug dispersal into cotton probably because it was approximately the same height as peanut. In 2012, flowering buckwheat increased the efficacy of *Trichopoda pennipes* (F.) attacking *N. viridula* in cotton although it did not deter dispersal of stink bugs. In conclusion, a barrier at least as tall as cotton can effectively retard the entry of stink bug adults into cotton.

## Introduction

The southern green stink bug, *Nezara viridula* (L.), the brown stink bug, *Euschistus servus* (Say), and the green stink bug, *Chinavia hilaris* (Say) (Heteroptera: Pentatomidae), are economic pests of cotton (Barbour et al. 1990; Turnipseed et al. 1995; Bundy and McPherson 2000). *Chinavia hilaris*, also known as *Acrosternum hilare* (Say), has been formally resolved to *C. hilaris* (Schwertner and Graziz 2007; Rider 2009). In the coastal plain of the southeastern USA, cotton is a mid-to-late-season host crop for these stink bug species (Bundy and McPherson 2000). Adult stink bugs colonize cotton to feed on fruit and oviposit on foliage (Tillman 2013). Feeding on bolls by adults and subsequent nymphs results in boll damage which can be assessed by examining a boll for internal injury (i.e., warts and stained lint) (Bundy et al. 2000).

*Nezara viridula* and *Euschistus* spp. move between closely associated host plant habitats within farmscapes throughout the growing season in response to deteriorating suitability of their host plants in these habitats (Toscano and Stern 1976; Velasco and Walter 1992). In the coastal plain of the southeastern USA, peanut and cotton are two crops common to farmscapes (i.e., multiple fields of different crops whose edges interface with each other and non-crop habitats). Raster maps of interpolated stink bug populations, spatial analysis by distance indices (SADIE) methodology (Perry et al. 1999), and mark-recapture studies demonstrated that *N*. *viridula* and *E. servus* adults that develop in peanuts disperse into cotton (Tillman et al. 2009). A recent study on colonization of *N. viridula*, *E. servus*, and *C. hilaris* in peanut-cotton farmscapes revealed that cotton was a relatively good host for all three stink bug species, but peanut, although a good host for nymphal development of *N. viridula* and *E. servus*, was a surprisingly poor host for *C. hilaris* (Tillman 2013). Nevertheless, adults of *C. hilaris* also exhibit edge-mediated dispersal at peanut-to-cotton interfaces as they colonize cotton, and density of each of the three stink bug species is significantly higher in cotton at these interfaces than in field edges adjacent to non-crop habitats (Tillman et al. 2014). An edge effect in dispersal of *C. hilaris* adults was detected in cotton adjacent to woodlands (Tillman et al. 2014) indicating that the non-crop host plants (Jones and Sullivan 1982) detected in woodlands were sources of this stink bug in cotton. Also, dispersal of this stink bug in a crop tended to increase as crop height decreased (Tillman et al. 2014). Therefore, the low growth of peanut likely facilitates flight of *C. hilaris* from non-crop host plants in woodlands across peanut and into cotton. Thus, strategic placement of a physical barrier, either synthetic or plant-based, at the peanut-to-cotton interface could manage these pests. Indeed, the fact that these stink bugs are known to disperse into cotton at these interfaces provides an excellent opportunity to evaluate the ability of a physical barrier to retard the entry of colonizing stink bugs into crops in general.

In this study, both sorghum sudangrass and buckwheat were examined as potential plant-based barriers to dispersal of stink bugs into cotton. Flowers of buckwheat, though, secrete nectar composed of sucrose, fructose, and glucose (Cawoy et al. 2008). *Trichopoda pennipes* (F.) (Diptera: Tachinidae) parasitizes *N. viridula* in a variety of crops (Todd and Lewis 1976; McPherson et al. 1982; Menezes et al. 1985; Tillman 2006a; Tillman 2008). Nectar can impact the fecundity and longevity of *Trichopoda* spp.; for example, all female *T. giacomellii* (Blanchard), a South American parasitoid of *N. viridula*, provided with only water died within 3–4 days of emergence and produced approximately 20 % of the eggs of females provided raisins (Coombs 1997). When buckwheat was incorporated in the farmscape in an earlier study, parasitism rates by *Cotesia rubecula* (Marshall) on imported cabbage worm [*Pieris rapae* (L.)] larvae were increased (Lee and Heimpel 2005).

The current study is an investigation into the effectiveness of a physical barrier, either synthetic or plant-based, at the peanut-to-cotton interface to suppress the entry of stink bug adults into cotton plots. The ability of buckwheat to enhance parasitism of *N. viridula* by T*. pennipes* in cotton at the peanut-cotton interface also was examined.

## Materials and methods

### Study site

In 2012, the experiment was conducted in an on-farm peanut-cotton farmscape (31°32′30.18″N, 83°17′42.03″N) in Irwin County, GA, USA. The next year, it was conducted in the same county but at another site (31°33′29.98″N, 83°17′47.65″W). Rows were planted 0.9 m apart for each crop. Peanut (Birdsong GA-06G) was planted on 18 May 2012 and 25 May 2013. Fibermax 499 cotton was planted on 1 June 2012, and Deltapine 1137 cotton was planted on 21 May 2013. Sorghum sudangrass (Super Sugar) was planted on 18 May 2012 and on 25 May 2013. In 2012, buckwheat was planted on 14 and 28 June and 13 and 27 July. Multiple planting dates for buckwheat ensured continuous presence of mature plants while cotton was fruiting.

### Sampling procedures

Peanut and cotton were examined for presence of *N. viridula*, *C. hilaris*, and *E. servus* during the growing season. The peanut canopy within a 7.3 m length of row of was swept (sweep net 38 cm in dia.) to capture stink bugs. Cotton sampling began with the first presence of fruit (i.e., bolls). For each cotton sample, all plants within a 1.8 m length of row were shaken over a drop cloth and visually examined for stink bugs. Boll damage was assessed by examining a boll (≈2.5 cm in dia.) at each sample for internal injury caused by stink bugs as described by Bundy et al. (2000). The treatment threshold is set at 20 % internal boll injury during the 2nd week of bloom, 10–15 % internal boll injury during the 3rd through 5th weeks of bloom, 20 % during the 6th week of bloom, and 30 % during the 7th week of bloom (Bacheler et al. 2009). Stink bug adults collected during sampling were held for parasitoid emergence. In 2012, buckwheat was visually examined for nectar feeding by stink bug parasitoids. Voucher specimens of stink bugs and *T. pennipes* are held in the USDA, ARS, Crop Protection & Management Research Laboratory in Tifton, GA.

### 2010 Experiment

**Fig. 1:**
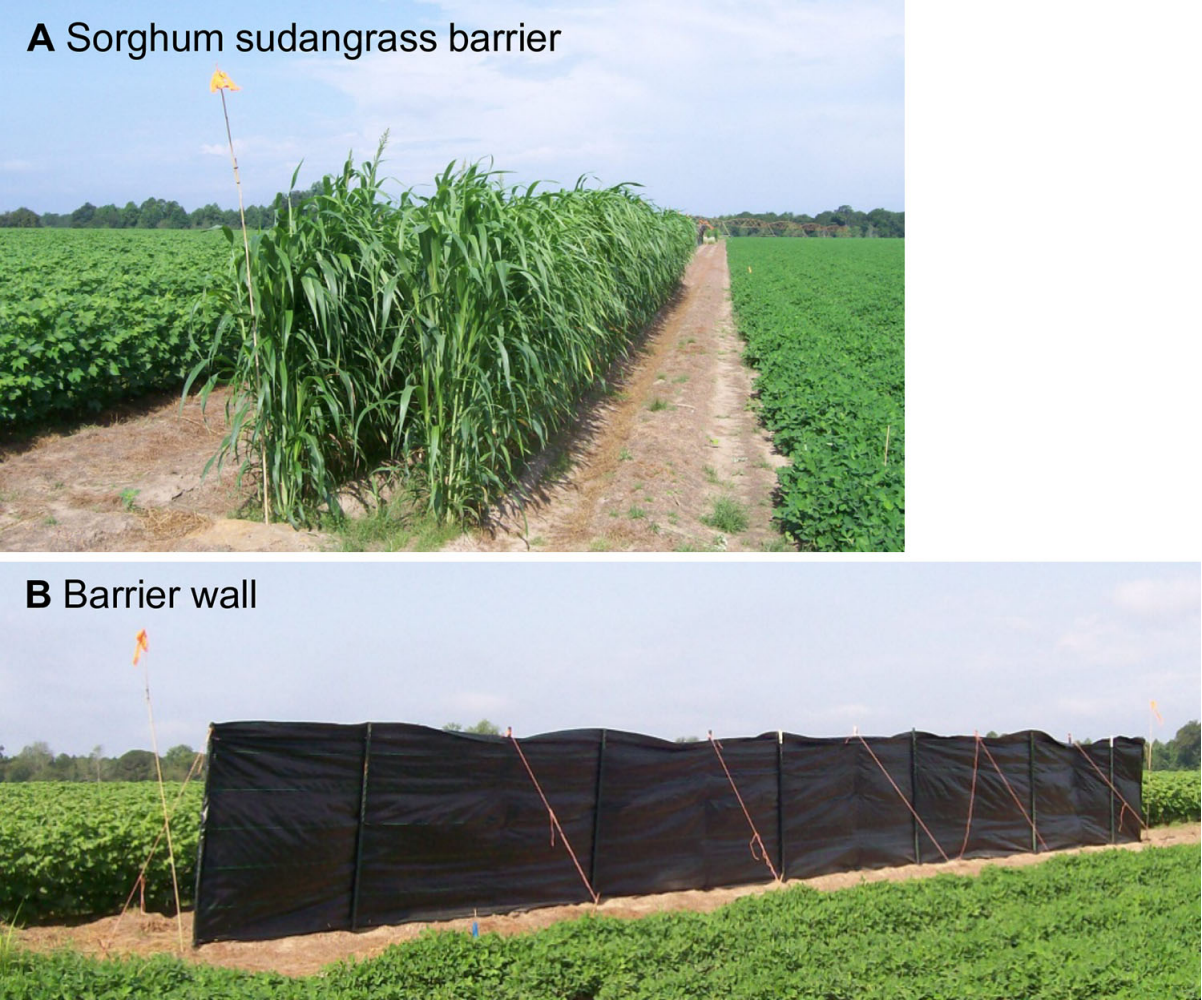
Photo of the sorghum sudangrass barrier (**a**) and 1.8-m-high barrier wall **(b)** between peanut and cotton in Irwin County, GA, USA

All treatment plots were established in the peanut-cotton farmscape at the interface of the two crops. Each plot was 22.9 m along the interface and 64.0 m wide (i.e., 70 rows); cotton and peanut were both 30.2 m wide (i.e., 33 rows), and the various treatments were established in four rows (3.6 m wide) between cotton and peanut plots. The five treatments were as follows: (1) a 1.8-m-high barrier wall, (2) a 0.6-m-high barrier wall, (3) two rows of sorghum sudangrass (Fig. 1a), (4) two rows of buckwheat, and (5) a control. A barrier wall consisted of a 22.9-m-wide sheet of black ≈85 g UV stabilized polypropylene supported by T-style metal fence posts (Fig. 1b). Each treatment was randomly assigned to a plot within a block for each of four blocks (replicates) in a randomized complete block design (RCBD). Peanut and cotton were sampled weekly for 8 weeks beginning on 24 July. In the previous study, an edge effect in dispersal of stink bugs into cotton was detected up to 8.2 m (row 9) from the crop-to-crop interface in peanut-cotton farmscapes (Tillman et al. 2014). So, for both crops for both years of the study, samples were obtained from rows 1 (0.9 m), 2 (1.8 m), 5 (4.6 m), 9, 16 (14.6 m), and 33. In 2012, one sample was obtained per row sampled in peanut, while six samples were taken per row sampled in cotton. Mature plant height for peanut, cotton, buckwheat, and sorghum sudangrass was measured for ten randomly selected plants per crop per treatment replicate.

### 2013 Experiment

Plot size and location were as described for the 2012 experiment. The five treatments were: (1) a 1.8-m-high barrier wall, (2) a 1.2-m-high barrier wall, (3) a 0.6-m-high barrier wall, (4) two rows of sorghum sudangrass, and (5) a control. Each treatment was randomly assigned to a plot within a block for each of four blocks in a RCBD. Preliminary examination of the 2012 data revealed that buckwheat was a very unsuitable physical barrier to dispersal of stink bugs into cotton, and thus this treatment was replaced by a 1.2-m-high barrier wall in 2013. Peanut and cotton were sampled weekly for 5 weeks beginning on 30 July. One sample was obtained per row sampled in peanut, while three samples were taken per row sampled in cotton. Mature plant height for peanut, cotton, and sorghum sudangrass was measured for ten randomly selected plants per crop per treatment replicate.

### Analysis

Stink bug density data in peanut and cotton were analyzed using PROC MIXED (SAS Institute Inc 2008). For both years of the study, the first sampling date was excluded from analyses due to the absence of stink bugs in cotton in 2012 and the presence of only six stink bugs in this crop in 2013 on this sampling date, and row 33 was excluded from data analyses due to the absence of stink bugs on this row. Fixed effects were week, treatment, row sampled, week by treatment, week by row, treatment by row, and week by treatment by row. Random effects were replicate and residual error. In 2012, buckwheat was included in the barrier test as a potential barrier to stink bug dispersal. Observations of *T. pennipe*s in the field, though, revealed that this parasitoid readily fed on nectar of buckwheat. So, 2012 data for parasitism rates of *N. viridula* by *T*. *pennipes* were analyzed using PROC MIXED (SAS Institute Inc 2008). Preliminary analyses revealed that none of the week and row effects for parasitism rates of stink bugs were significant, and so the data were analyzed using treatment as the fixed effect. Arcsine square-root transformation was used to normalize percentage parasitism data. Preliminary analyses for peanut revealed that none of the week and row effects for stink bug density were significant, and so the data were analyzed using treatment as the fixed effect. Square-root transformation was used to normalize stink bug data. Mature plant height data was analyzed using PROC MIXED (SAS Institute Inc 2008). The fixed effect was crop. Least squares means were separated by the least significant difference (SAS Institute Inc 2008) where appropriate.

## Results

### 2012 Experiment

**Fig. 2:**
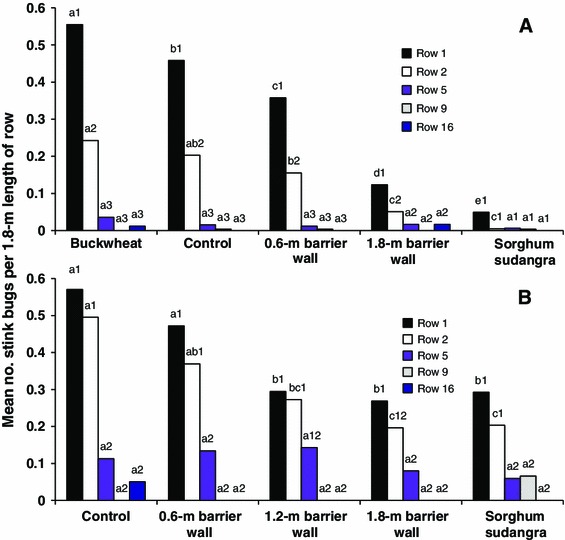
Least squares means for number of stink bugs per 1.8 m length of row in cotton rows in the barrier experiment in 2012 (**a**) and 2013 (**b**). Means followed by the same *lowercase letter* are not significantly different between treatments for a single row, and means followed by the same *number* are not significantly different between cotton rows for a single treatment (LSD, *P* > 0.05, common SE = 0.0248 in 2012, common SE = 0.0475 in 2013)

In cotton, the percentage of *E. servus*, *N. viridula*, and *C. hilaris* was 62.8, 36.4, and 0.8 %, respectively. Adult stink bug density in cotton was significantly influenced by week (*F* = 5.78, df = 6, 3425, *P* < 0.0001), treatment (*F* = 33.91, df = 4, 3425, *P* < 0.0001), row sampled (*F* = 141.45, df = 4, 3425, *P* < 0.0001), week by treatment (*F* = 1.92, df = 24, 3425, *P* < 0.0045), week by row (*F* = 3.39, df = 24, 3425, *P* < 0.0001), and treatment and row (*F* = 14.62, df = 16, 3425, *P* < 0.0001). There was no significant week by treatment by row interaction (*F* = 1.09, df = 96, 3425, *P* < 0.2616). Except for the sorghum sudangrass treatment, stink bug density was significantly higher on row 1 compared to all other rows sampled (Fig. 2a). For cotton row 1, stink bug density was significantly different between all barrier treatments; density was lowest for the sorghum sudangrass barrier and highest for the buckwheat treatment relative to all other treatments. Stink bug density was significantly higher on row 2 compared to rows 5, 9, and 16 for the buckwheat, control, and 0.6-m-high barrier wall treatments. For cotton row 2, stink bug density was significantly higher for the buckwheat and control treatments compared to the 1.8-m barrier wall and sorghum sudangrass treatments. Unlike on row 1, there was no significant difference in stink bug density between the control and the 0.6-m barrier wall on row 2. Overall, stink bug density was higher for buckwheat and control treatments compared to the higher barrier wall and sorghum sudangrass on cotton rows 1 and 2, while stink bug density for the shorter barrier wall was lower than or similar to the control over these two rows. Economic threshold was reached in only control cotton (all replicates) for week 8, but the cotton field did not receive an insecticide treatment.

**Fig. 3:**
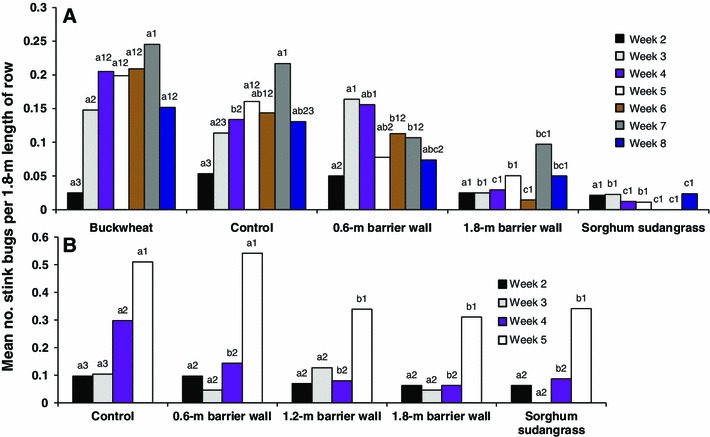
Least squares means for number of stink bugs per 1.8 m length of row in cotton each week in the barrier experiment in 2012 (**a**) and 2013 (**b**). Means followed by the same *lowercase letter* are not significantly different between treatments for a single week, and means followed by the same *number* are not significantly different between weeks for a single treatment (LSD, *P* > 0.05, common SE = 0.0278 in 2012, common SE = 0.0424 in 2013)

On week 2, there was no significant difference in stink bug density between barrier treatments (Fig. 3a). However, with one exception, stink bug density was significantly higher for the buckwheat and control treatments compared to the 1.8-m-high barrier wall and sorghum sudangrass treatments for each remaining sampling week. These results are similar to those for rows 1 and 2 for these treatments (Fig. 2a). On week 8, there was no significant difference in stink bug density between the control and the 1.8-m barrier wall (Fig. 3a). On weeks 3, 4, and 6, stink bug density was significantly higher for the 0.6-m barrier treatment relative to the 1.8-m barrier wall and sorghum sudangrass treatments. However, on weeks 5 and 8, there was no significant difference in stink bug density between the 0.6-m barrier wall treatment and the 1.8-m barrier and sorghum sudangrass treatments. For the treatments with the overall highest stink bug density, the buckwheat and control treatments, density was relatively higher for weeks 5, 6, and 7 than for the other sampling weeks.

**Fig. 4:**
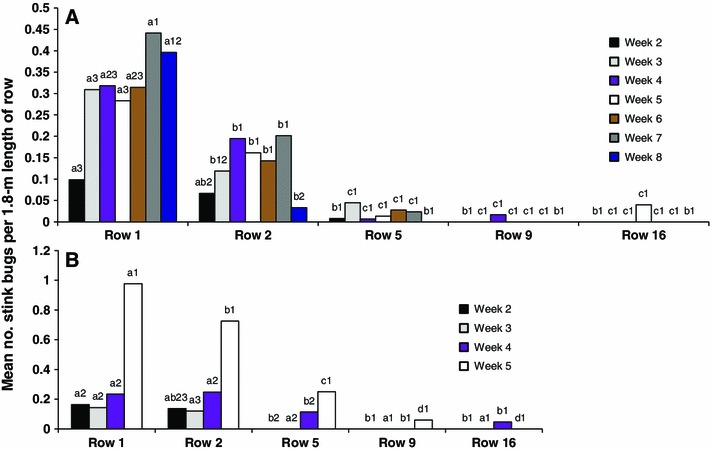
Least squares means for number of stink bugs per 1.8 m length of row in cotton rows each week in the barrier experiment in 2012 (**a**) and 2013 (**b**). Means followed by the same *lowercase letter* are not significantly different between rows for a single week, and means followed by the same *number* are not significantly different between weeks for a single row (LSD, *P* > 0.05, common SE = 0.0278 in 2012; common SE = 0.0424 in 2013)

Except for weeks 2 and 8, stink bug density was significantly higher on row 1 relative to all other rows and higher on row 2 compared to rows 5, 9 and 16 (Fig. 4a) similar to the results for rows for the buckwheat, control, and 0.6-m-high barrier wall treatments (Fig. 2a). On row 1, stink bug density was significantly higher on week 7 compared to weeks 2 though 6 (Fig. 4a).

In peanut, the percentage of *E. servus*, *N. viridula*, and *C. hilaris* was 63.3, 30.0, and 6.7 %, respectively. In this crop, stink bug density was not influenced by treatment for both nymphs (*F* = 0.95, df = 4, 795, *P* = 0.43, common SE = 0.0415) and adults (*F* = 1.56, df = 4, 795, *P* = 0.18, common SE = 0.0213). Thus, treatment differences in cotton were not due to variable densities of stink bugs in peanut.

There were significant differences in mature height of plants (*F* = 4178.2, df = 3, 475, *P* < 0.0001, common SE = 0.0146). Mature plant height was significantly higher for sorghum sudangrass (2.4 m high) than for cotton (1.4 m high), higher for cotton than for buckwheat (0.6 m high), and higher for buckwheat than for peanut (0.4 m high).

**Fig. 5:**
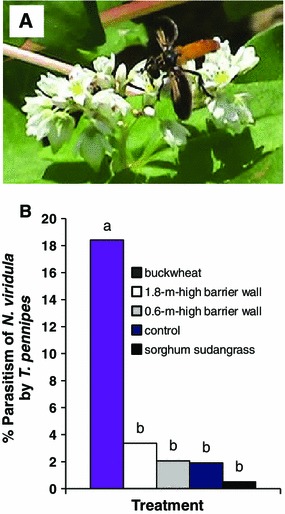
Photo of a *T. pennipes* adult feeding on nectar of buckwheat (**a**) and least squares means for percentage parasitism of *N. viridula* adults by *T. pennipes* in cotton adjacent to buckwheat, sorghum sudangrass, a 1.8-m high and 0.6-m high barrier wall, and a control in 2012 (**b**). Means followed by the same letter are not significantly different between treatments (LSD, *P* > 0.05, common SE = 1.98)

Throughout the experiment, the stink bug adult parasitoid *T. pennipes* was observed feeding on nectar of buckwheat (Fig. 5a). Percentage parasitism of *N. viridula* adults by *T. pennipes* in cotton was significantly higher in cotton adjacent to buckwheat compared to any other cotton treatment (Fig. 5b; *F* = 14.22, df = 4, 111, *P* < 0.0001).

### 2013 Experiment

In cotton, the percentage of *E. servus*, *N. viridula*, and *C. hilaris* was 82.2, 15.7, and 2.1 %, respectively. Adult stink bug density in cotton was significantly affected by week (*F* = 70.17, df = 3, 1096, *P* < 0.0001), treatment (*F* = 6.81, df = 4, 1096, *P* < 0.0001), row sampled (*F* = 57.14, df = 4, 1096, *P* < 0.0001), week by treatment (*F* = 1.99, df = 12, 1096, *P* < 0.0221), week by row (*F* = 17.28, df = 12, 1096, *P* < 0.0001), and treatment and row (*F* = 2.23, df = 16, 1096, *P* < 0.0036). There was no significant week by treatment by row interaction (*F* = 1.01, df = 48, 1096, *P* < 0.4537). Except for the 1.2-m-high barrier wall treatment, stink bug density in cotton was significantly higher on row 1 compared to rows 5, 9, and 16 (Fig. 2b). For the 1.2-m barrier wall, there was no significant difference in stink bug density between rows 1, 2, and 5. Stink bug density was significantly higher on row 2 compared to rows 5, 9, and 16 for the control, 0.6-m-high barrier wall, and sorghum sudangrass treatments. There was no significant difference, though, in stink bug density between row 2 and rows 5, 9, and 16 for the 1.8-m-high barrier wall. For rows 1 and 2, stink bug density was significantly higher for the control and 0.6-m-high barrier treatments compared to the 1.8-m-high barrier wall and sorghum sudangrass treatments. On row 1, stink bug density was significantly lower for the 1.2-m-high barrier wall compared to the control and the shortest barrier wall, but on row 2, there was no significant difference in stink bug density for the 1.2-m-high barrier wall compared to the shortest barrier wall. For rows 5, 9, and 16, there was no significant difference in stink bug density between treatments. Economic threshold was reached in only control cotton (all replicates) and cotton next to the 0.6-m-high barrier wall (all replicates) for 1 week; the grower choose to apply dicrotophos at a rate of 292.3 ml/ha to the whole cotton field for stink bug control.

Stink bug density was significantly higher on week 5 relative to all other weeks for all treatments (Fig. 3b). On week 5, stink bug density was significantly higher for the control and 0.6-m-high barrier wall compared to the 1.2-m and 1.8-m high barrier wall and sorghum sudangrass. This was similar to the results observed for stink bug density on row 1 for the barrier treatments (Fig. 2b). On week 4, stink bug density was significantly higher for the control relative the remaining treatments (Fig. 3b). There was no significant difference between treatments on weeks 2 and 3.

On weeks 4 and 5, stink bug density was significantly higher on row 1 and 2 relative to all other rows (Fig. 4b) similar to the results for stink bug density on these two rows in comparison to the other rows for the control, shortest barrier wall, and sorghum sudangrass (Fig. 2b). On week 5, when stink bug density was at its highest level, density was higher for row 1 compared to row 2, higher for row 2 compared for row 5, and higher for row 5 compared to rows 9 and 16 (Fig. 4b).

In peanut, the percentage of *E. servus*, *N. viridula*, and *C. hilaris* was 84.6, 11.6, and 3.8 %, respectively. In this crop, stink bug density was not influenced by interface treatment for both nymphs (*F* = 2.19, df = 4, 589, *P* = 0.07, common SE = 0.0157) and adults (*F* = 1.28, df = 4, 588, *P* = 0.28, common SE = 0.0209) so differences in stink bug density detected in cotton were not due to differences in stink bug density in peanut.

There were significant differences in mature height of plants (*F* = 1,696.2, df = 2, 436, *P* < 0.0001, common SE = 0.0195). Mature plant height was significantly higher for sorghum sudangrass (2.1 m high) than for cotton (1.3 m high), and higher for cotton than for peanut (0.5 m high).

## Discussion

Unsurprisingly, stink bug density can increase over time and across rows. The data, though, clearly show that the number of adult stink bugs entering cotton plots at the peanut-cotton interface can be significantly reduced by the presence of a physical barrier, either synthetic, especially the 1.8-m-high barrier wall, or plant-based, especially sorghum sudangrass. Only one reference has been found on the evaluation of physical barriers for managing stink bug adults. Recently, Grasswitz and Fimbres (2013) determined that bagging peach fruit with Maggot Barrier® nylon mesh bags significantly reduced the percentage of peaches damaged by stink bugs but increased the percentage of fruit with skin marks. A thin mesh physical barrier around the peach orchard may be as effective in reducing damage to fruit as the mesh bags surrounding the fruit without marking the skins marks. The current study is the first published study in which physical barriers have been evaluated as a management tool for stink bugs in cotton. Physical barriers have been evaluated for other insect pests. An earlier study demonstrated that a plastic trench was an effective barrier to Colorado potato beetles, *Leptinotarsa decemlineata* (Say), as they walked into potato fields (Boiteau et al. 1994). The root weevils *Barypeithes pellucidus* Boheman and *Nemocestes incomptus* Horn can be also effectively controlled in strawberry by plastic and aluminum fences treated with Teflon (Bomford and Vernon 2005). In Peru, crop damage caused by the Andean potato weevil, *Premnotrypes suturicallus* Kuschel was reduced significantly using plastic barriers (Kroschel et al. 2009). Plots of wheat or bare ground that were surrounded by similar trenches had significantly fewer click beetle, *Agriotes obscurus* L., males in pheromone traps and pitfall traps relative to non-trenched plots (Vernon and van Herk 2013).

Unlike the coleopteran adults mentioned above that walk into new crops, colonizing adult stink bugs can fly. However, in the current study, they primarily moved across control cotton rows close to peanut. Earlier studies support this edge effect in distribution of stink bugs as they colonize new crops (Espino et al. 2008; Toews and Shurley 2009; Reay-Jones 2010; Olson et al. 2011; Tillman et al. 2014). Likely, this edge effect is due to the fact that adult stink bugs tend to move along a path of the least resistance, along rows rather than across them (Panizzi et al. 1980; Tillman et al. 2009). The success of the physical barriers in the current study likely is due in part to this edge effect in distribution of stink bugs in crops.

Along field edges, plant height can influence dispersal of stink bugs into a crop, with taller plants serving as barriers to dispersal or channeling stink bugs along an edge (Tillman et al. 2014). In this study, a physical barrier at least as tall as cotton effectively retarded the entry of stink bug adults into cotton plots. Therefore, the success of the 1.8-m-high barrier wall and sorghum sudangrass barrier in protecting cotton apparently was due in part to the disruption of the flight of adult stink bugs from low-growing peanut into cotton.

One benefit of using a physical barrier to manage stink bugs could include a reduction in usage of insecticides for control of these pests and subsequent conservation of natural enemies. For both years of the study, stink bug damage reached economic threshold in only control cotton plots and cotton plots near the barrier wall approximately the same height as peanut indicating that utilizing physical barriers taller than cotton for management of stink bugs could lead to reduced insecticide applications. Under field conditions, insecticides commonly used to control stink bugs likely are also highly toxic to stink bug parasitoids, for laboratory studies have revealed that dicrotophos, oxamyl, and cyfluthrin are equally toxic to *N. viridula* and its parasitoid *T. pennipes* in residual and oral bioassays (Tillman 2006b). Clearly, stink bug parasitoids could be conserved by reductions in usage of these insecticides.

Incorporation of a nectar-producing plant adjacent to cotton increases the efficacy of *T. pennipes* attacking *N. viridula* in this crop. Although buckwheat did not deter dispersal of stink bugs into cotton, percentage of parasitism of *N. viridula* by *T. pennipes* was increased by incorporating this nectar-producing plant adjacent to cotton in 2012. Approximately, 1.6 km from the 2012 site, parasitism of primarily *N*. *viridula* by *T. pennipes* was significantly higher in cotton when the nectar-producing plant bloodflower, *Asclepias curassavica* L. (Apocynaceae), was planted next to the crop at a peanut-cotton interface in 2009 and 2010 (Tillman and Carpenter 2014). Other studies have demonstrated an increase in biological control of insect pests in the presence of nectar-producing flowers (Ellis et al. 2005; Lavandero et al. 2005; and Irvin et al. 2006). Buckwheat, though, is easy to establish, and nectar production attracts numerous other insect parasitoid species, as well as insect pollinators (Bowman et al. 2012). The two management tactics, physical barriers and incorporation of nectar-producing plants, are highly compatible with each other and could enhance conservation biological control of natural enemies and protect insect pollinators primarily by reduction in applications of insecticides.

Although significant suppression of these stink bugs using physical barriers was demonstrated in this study, the full potential of physical barriers as a practical option has yet to be determined. Placement and size of physical barriers to retard entry of stink bugs into crops could vary depending on landscape composition including crop types and adjoining stink bug reservoir habitats and landscape structure such as position of crops, crop height, and height of source plants within reservoir habitats. However, the paucity of effective alternative control measures available for stink bug management, especially in organic cropping systems, justifies further full-scale evaluations into physical barriers for control of these pests in crops.
